# Social, microbial, and immune factors linking bacterial vaginosis and infectious diseases

**DOI:** 10.1172/JCI184322

**Published:** 2025-06-02

**Authors:** Nicole M. Gilbert, Luis A. Ramirez Hernandez, Daniela Berman, Sydney Morrill, Pascal Gagneux, Amanda L. Lewis

**Affiliations:** 1 Department of Pediatrics,; 2 Center for Women’s Infectious Disease Research,; 3 Department of Molecular Microbiology, and; 4 Department of Obstetrics and Gynecology, Washington University School of Medicine, St. Louis, Missouri, USA.; 5 Department of Obstetrics, Gynecology, and Reproductive Sciences,; 6 Glycobiology Research and Training Center,; 7 Department of Pediatrics, and; 8 Departments of Pathology and Anthropology, UCSD, La Jolla, California, USA..

## Abstract

Bacterial vaginosis (BV) is a polymicrobial condition of the vaginal microbiota associated with a variety of sexually transmitted infections, infections of maternal and fetal tissues during pregnancy, and even some infections outside of the reproductive tract, including the urinary tract and mouth. BV has also been associated with conditions in which the body generates prominent inflammatory reactions to microbes, including infections of the cervix and other upper genital tract tissues. For reasons still not understood, BV is a highly recurrent and often difficult-to-treat condition, complicating attempts to prevent these associated infections. An additional layer of complexity arises from the increasing awareness that the presence of BV-associated bacteria in the vagina is not always symptomatic or associated with adverse outcomes. In this concise Review, we summarize and synthesize three groups of factors grounded in the literature that may be fueling the associations between BV and infection: (a) aspects of society and culture; (b) pathogens, virulence factors, and processes of microbial antagonism and synergy; and (c) host factors, such as genetics and immunity. Our goal is to understand what contexts and combinations of microbial, host, and social factors conspire to make BV virulent in some individuals but not others. Disrupting these patterns more systematically may achieve healthier outcomes.

## Introduction

In the following Review, we briefly discuss the overlapping set of sociocultural, microbial, and host-intrinsic factors that are associated with bacterial vaginosis (BV) and with infection. Our aims are to provide a context for these associated factors based on existing literature and to create a framework for further comprehensive and systematic analysis of the relationship between BV and infection.

## Contours of the vaginal microbiome in health and disease

### Lactic acid bacteria.

The dominance of *Lactobacillus* species in the vagina of reproductive-age women and associated acidic milieu, apparently unique to the human vagina (1), has been associated with lower risks of adverse health outcomes such as preterm birth (2, 3). Vaginal *Lactobacillus* species are believed to thwart colonization of potential pathogens, largely by producing lactic acid and establishing the vagina’s acidic pH (4). To accomplish this, lactobacilli have active mechanisms of resistance to acid stress not shared by many other microbes (5). Five major community state types (CSTs) of the vaginal microbiota have been described in asymptomatic women; four of these have different species of *Lactobacillus* as their dominant members. The two most common *Lactobacillus*-dominant CSTs are characterized by either *Lactobacillus crispatus* (CST-I) or *Lactobacillus iners* (CST-III). Other *Lactobacillus* species can inhabit the vagina, for example *L*. *gasseri* (CST-II) and *L*. *jensenii* (CST-V). But these are less common as the dominant taxa (6). Finally, CST-IV is not a *Lactobacillus*-dominant microbiota but instead is categorized by higher α diversity and many other signature features (see below).

### Lactobacillus-deplete microbial signatures.

A vaginal microbiome with a polymicrobial community of diverse bacteria and low or no *Lactobacillus* has been referred to by the terms bacterial vaginosis, CST-IV, or molecular BV, each of which reflect different (and sometimes inconsistent) methodological approaches (6–9). Nearly one-third of reproductive-age US women meet criteria for BV, but rates vary between women of different ages and racial or ethnic backgrounds (10–13). It is increasingly recognized that racialized terms with minimal cultural or geographic specificity (e.g., “Black,” “Hispanic”) are scientifically invalid as biological categories (14). However, as noted later, such language may reflect sociocultural realities that can be more challenging to define and measure. That said, in early studies of reproductive-age women, about 40% of women identifying as Black or Hispanic had diverse BV-like vaginal communities compared with 10% of women identifying as White (6). Black and Hispanic individuals also had higher vaginal pH (4.7–5.0) compared with those identifying as Asian or White (pH 4.2-4.4) (6).

Longitudinal studies, especially those involving molecular tools, have revealed that extrinsic factors, like sexual activity and antibiotic treatments, as well as intrinsic factors like menarche, menses, and menopause, can be associated with dramatic shifts in individual microbial dynamics (15–20). Despite shifts in many of these factors, the categorical CST classification in reproductive-age women remained relatively stable over time in about 75% of individuals (9).

Individuals carrying a CST-IV microbiome may not experience or self-report vaginal symptoms, leading to a reasonable interpretation that a diverse vaginal microbiome can be a “normal” or “healthy” state in some individuals some of the time. Despite existing in many asymptomatic individuals, BV is one of the most common symptomatic vaginal conditions that prompt clinician visits. Symptoms of BV can include changes in the amount or consistency of vaginal discharge or a fishy odor. In the clinic, BV diagnosis is made by meeting three of four Amsel criteria: abnormal discharge, pH > 4.5, >20% of epithelial cells are clue cells (cells coated with adherent bacteria), and an amine odor upon addition of potassium hydroxide (the “whiff” test) (8). A different laboratory-based method, Nugent scoring, relies on microscopic scoring of Gram-stained bacterial morphotypes to determine BV status (21). The Amsel and Nugent methods are effective for classification but crude. They do not always agree with each other or with other molecular methods of characterizing the vaginal microbiota (6, 22, 23).

Whether referred to as CST-IV or BV, studies agree that these microbiotas contain a greater diversity of microbes than all other CSTs. These include *Gardnerella*, *Fannyhessea* (previously known as *Atopobium vaginae*), *Prevotella*, *Sneathia*, and other taxa. *L*. *iners* is often also present in these communities and is the only species of *Lactobacillus* found in abundance during BV/CST-IV. The CST-IV signature has considerable but imperfect overlap with the community of bacteria described by other methods as BV (6).

### The vaginal microbiome, BV, and infectious diseases.

**Figure 1:**
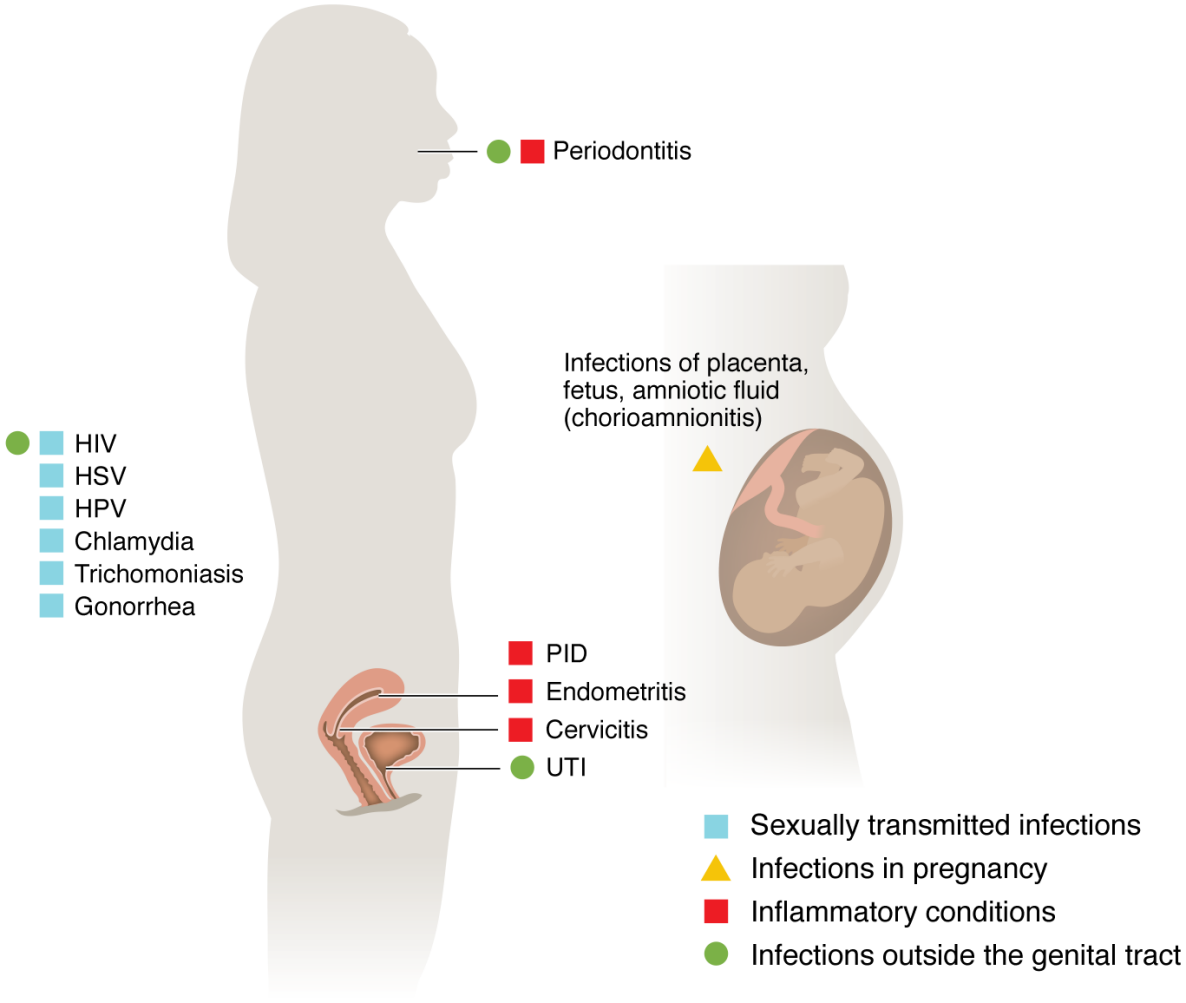
Infections associated with bacterial vaginosis fall into four main categories. These include sexually transmitted infections (examples indicated by blue squares), infections in pregnancy (yellow triangle), inflammatory conditions often associated with infections (red squares), and infections outside the genital tract (green circles). For our purposes, “inflammatory conditions” refers to diagnoses that are based on the requirement of gross clinical or histological inflammation, rather than defining a particular pathogen. However, these conditions are often infectious in nature, sometimes characterized by fever (e.g., chorioamnionitis, tubo-ovarian abscess), or calling for the use of antimicrobials (e.g., strawberry cervix, other PID) or tissue debridement (e.g., periodontitis) as standard of care.

**Figure 2:**
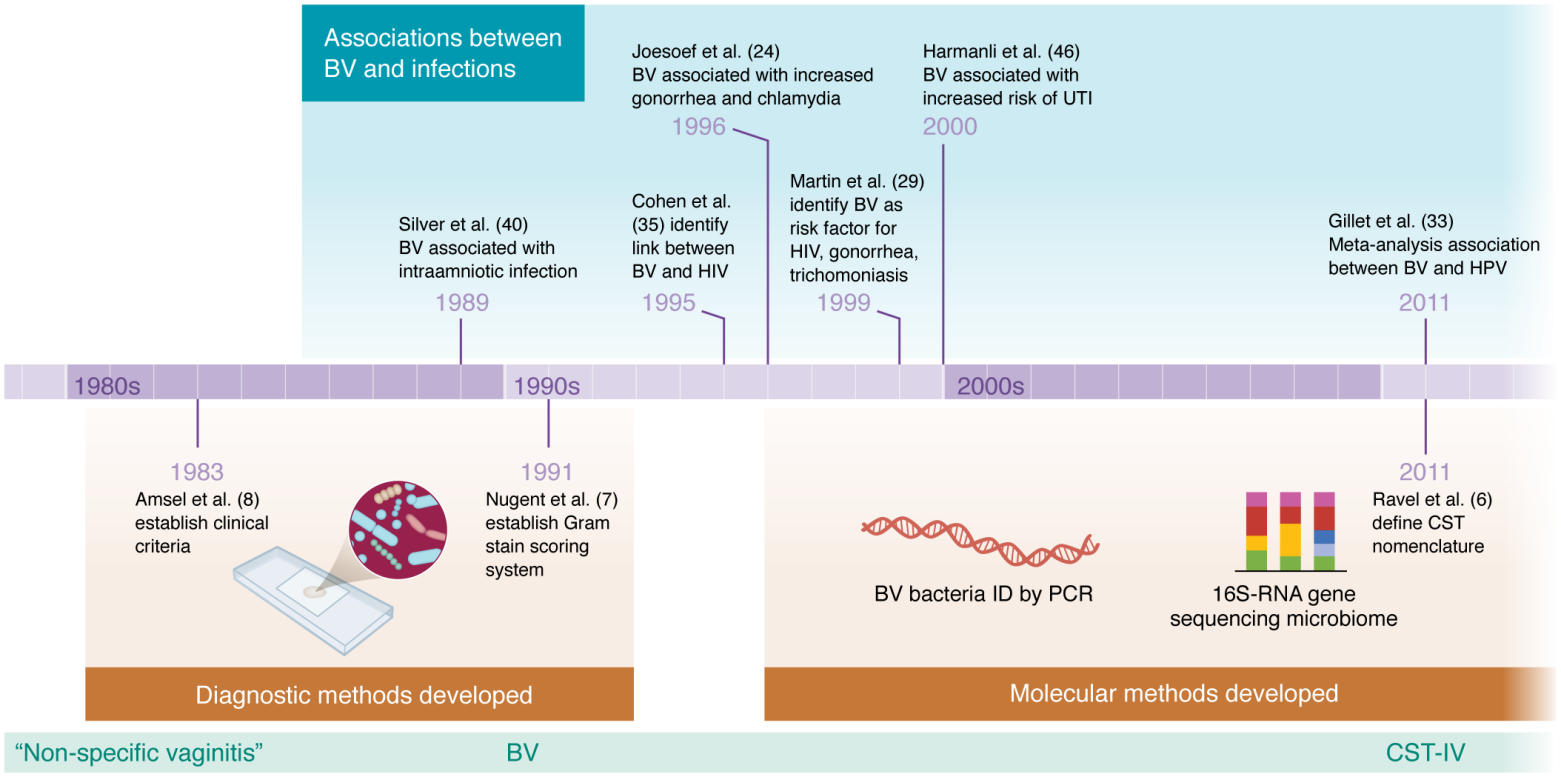
A brief history of advances contributing to the recognition of links between BV and infection. We have selected studies representing major milestones in the field. References correspond to the text, and several are summarized in Table 1. BV, bacterial vaginosis.

**Figure 3:**
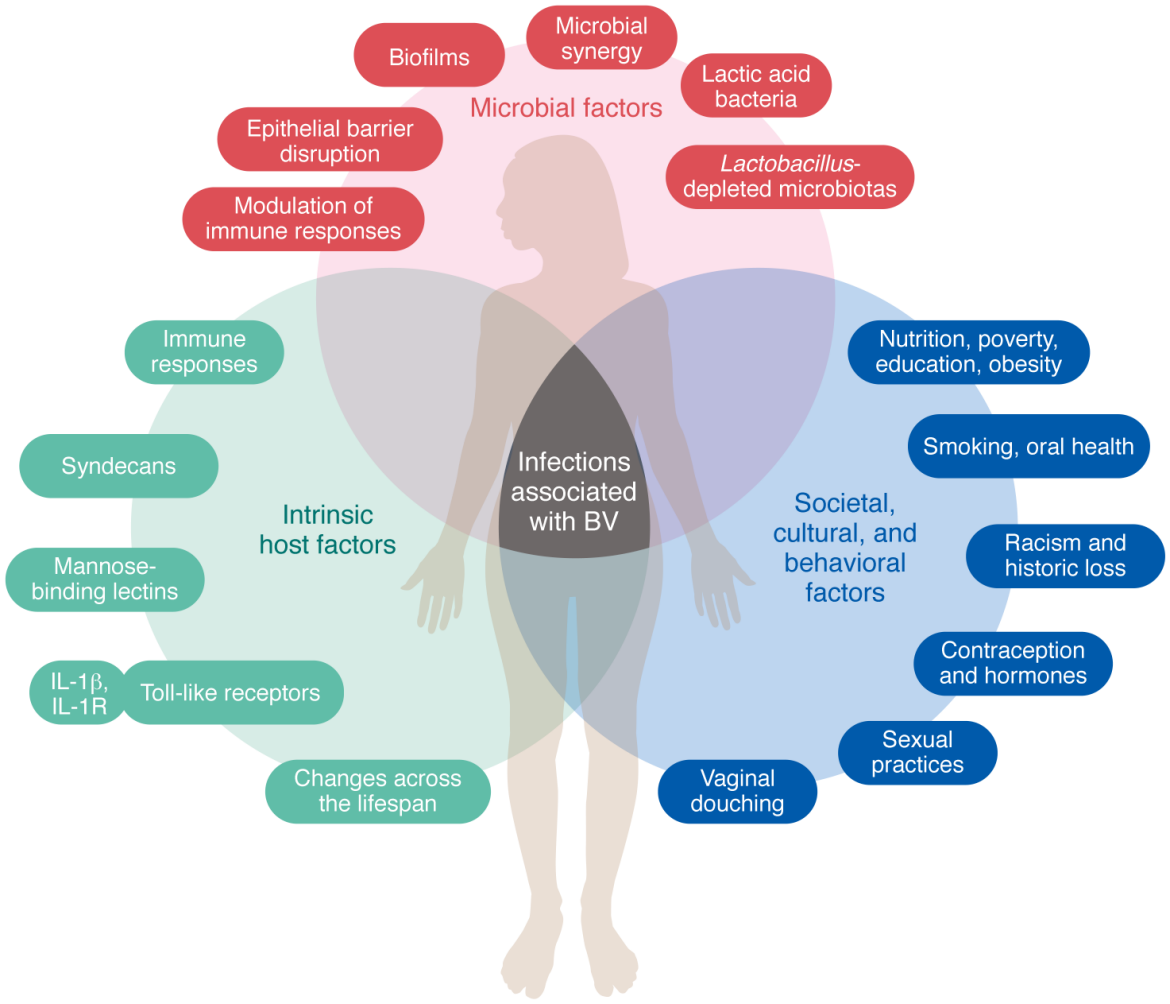
Three constellations of factors link the varied relationships between BV and infection. In the schematic, microbial factors, intrinsic host factors, and societal, cultural, and behavioral factors are defined as overlapping risk zones for BV and related infections.

Even though a diverse CST-IV vaginal microbiome can be asymptomatic, numerous studies over the past several decades have reported associations between BV and serious health problems — in particular a risk of many types of infections (Figure 1 and Table 1). BV has been identified as a risk factor in at least four different classes of infections we will discuss in this Review: (a) sexually transmitted infections (STIs), (b) infections specific to pregnancy, (c) conditions diagnosed by inflammation, and (d) infections outside the genital tract (Figure 1). This is not a systematic Review. However, the literature strongly supports a positive association between BV and infections by several bacterial, protozoan, and viral STIs, including chlamydia (11, 24–28), gonorrhea (11, 24–29), trichomoniasis (11, 28–30), human papillomavirus (HPV) (31–33), HIV (29, 34, 35), and herpes simplex virus (HSV) (36) (see timeline in Figure 2). In pregnancy, BV was associated with higher risks of infections of the placenta and amniotic fluid (37–42). BV has also been linked to other conditions in the female genital tract defined clinically by inflammation but often triggered by microbes (43), including infections of the cervix, endometrium (44), and other sites (45). Infections associated with BV are not limited to the genital tract, sometimes making a potential causal explanation more challenging, as for instance, in the context of BV’s relationships to urinary tract infections (UTIs) (44, 46–48) or periodontitis (49, 50) (Figure 1). In this Review, we focus on the links between BV and infection. We acknowledge that BV, or a CST-IV vaginal microbiome, is not always associated with infection, that certain associations are more or less apparent in different groups studied, and that association never proves causation. Why some women with BV experience symptoms or adverse outcomes while other women with a seemingly similar polymicrobial vaginal microbiota have no apparent adverse effects is an important question with few comprehensive answers. This Review focuses on three conceptual topic areas (Figure 3), discussing relevant factors potentially contributing to the links between BV and infection: (a) impacts of society, culture, behavior, and other extrinsic factors; (b) microbial factors and host responses to them; and (c) genetics and intrinsic host factors across the lifespan.

## Society, culture, and behavior

### Social determinants of health in BV.

Here, we synthesize literature reports describing associations between BV and social determinants of health, like socioeconomic status, educational attainment, adverse life experiences, nutritional status, obesity, and oral health. As discussed below, the literature has revealed many correlations between compositional patterns of the vaginal microbiota, extrinsic life circumstances, and other complex sociocultural behaviors like smoking, vaginal douching, contraceptive choices, and sexual practices. This concise Review will explore how aspects of our society, culture, and behavior may interact with constellations of intrinsic host factors as well as microbial factors to generate conceptually overlapping risk zones that may help refine our understanding of the interaction between the vaginal microbiota and infection.

### Nutritional status.

A growing body of evidence supports a correlation between low nutritional status, as well as obesity, with higher risks of BV and coincident infections. In a study of Indigenous women in North Queensland, Australia, poor nutritional status and a lack of folate were both associated with a higher risk of STIs and PID (51). Women recruited from a gynecology clinic in Tehran, Iran, who consumed a diet high in vegetables, nuts, fish, olives, and olive oil had lower odds for BV. Those with a diet high in sugar, solid oils, sweets, and desserts, red meat, fried potato, refined grains, visceral meat, and sweet drinks had a higher odds of BV (52–54). Obesity is positively correlated with both BV and STIs (55, 56). Vitamin D deficiencies have been extensively correlated with higher risks of BV, *Chlamydia*, and HIV (57–61). However, a 24-week supplementation strategy with vitamin D after antibiotic treatment for BV was ineffective in reducing rates of BV recurrence after antibiotic treatment (62, 63). Ultimately, nutrition and obesity are multidimensional entities, and fully disentangling their effects on the vaginal microbiome from many other interrelated socioeconomic components remains a challenge.

### Smoking and oral health.

Smoking and poor oral health have broad negative effects on the host immune system and are associated with higher risks of many kinds of infections, including BV, trichomoniasis, HIV, HPV, chlamydia, PID, surgical site infections, periodontal disease, and infections during pregnancy (64–69). In one study, having a BV-like vaginal microbiome was associated with 25-times higher likelihood to report being a smoker compared with someone with a dominant *L*. *crispatus* microbiota (70). Smoking tobacco as well as poor oral and vaginal health was linked to lower household income and other markers of socioeconomic status (71, 72). These social issues compound an already challenging landscape of the directly damaging effects of tobacco and oral diseases on the human body. While the mechanisms underlying the link between BV, smoking, and oral health are not fully understood, studies have identified compounds from tobacco present in vaginal secretions (67). This supports the theory that these compounds could directly interact with host or bacterial cells in the vagina. Additional studies are needed to determine whether smoking cessation or oral health interventions impact the composition of the vaginal microbiota, particularly in combination with other BV treatment strategies.

From a microbiological standpoint, periodontitis has been correlated with BV (49) and with significantly greater abundances of BV-associated bacteria in the vagina (47). *Fusobacterium nucleatum* is ubiquitous in the human mouth and its relative abundance is higher during active gum disease (73). The bacterium is also found more commonly in the BV vagina (74) and is also a common cause of amniotic fluid infection (37, 75). Periodontitis has also been recognized as a risk factor for preterm birth and pregnancy loss (29, 48, 49). A study of *n =* 120 pregnancies measured higher numbers of *F*. *nucleatum* in subgingival biofilms and compromised oral health in those who delivered preterm compared with their full-term counterparts (73). In laboratory studies, *F*. *nucleatum* addition to minimally processed human vaginal bacterial communities promoted outgrowth of the prominent BV bacterium, *Gardnerella* (76). Previous studies identifying correlations between receptive oral sex and BV (77–80) could be related to orogenital transfer of *F*. *nucleatum* promoting the overgrowth of BV bacteria in the vagina (76). Future studies are needed to understand how sexual behaviors and oral health are related and to test if oral health interventions might improve sexual and reproductive health.

### Racism and historic loss.

There are several common risk factors for BV, STI (81), and some pregnancy-associated infections. For example, people with these infections are more likely to self-identify as Black or African American (6, 81, 82), have lower socioeconomic status and fewer educational opportunities, and are more likely to be exposed to chronic psychosocial stress and a higher number of adverse early life experiences compared with individuals who self-identify with other races (13, 83–85). To tease apart associations between BV and STI, in 2008 Peipert et al. used Cox regression to estimate the associations and synergy index between BV and STIs (86). Their results suggested that there were factors in addition to BV and race individually that were contributing to the synergistic association (synergy index of 3.38), suggesting that “social network or the consequences of socioeconomic position” could be playing a role. A notable study of Native American women showed that BV was significantly associated with the experience and intensity of historic loss, a syndrome defined as “a multigenerational factor involving slavery, forced removal from one’s land, legally ratified race-based segregation, and contemporary discrimination” (45). Other sections of this Review illustrate that chronic stress and higher numbers of adverse life experiences are also associated with BV, emphasizing that life circumstances rather than racial categories may underlie the different outcomes associated with BV.

### Contraceptives and hormones.

Studies of reproductive-age individuals suggest that hormonal contraception has been associated with a lower diversity of vaginal microbiota and significantly lower risks for BV, PID, and trichomoniasis, but this may not be true for other STIs (e.g., HSV2, chlamydia) (87, 88). Spermicides and copper T intrauterine devices have also been linked with steadily increasing rates of BV in users evaluated up to 180 days (89). See also *The vaginal microbiota over the lifespan* below.

### Sexual practices.

BV has been associated with a wide range of different STIs (Table 1). There is a strong resemblance in behavioral risk profiles between women with BV and those with STIs (10, 90). Shared risk factors include having higher numbers of lifetime sexual partners, having concurrent partners, and having a history of STI. (8, 10–13, 77, 91–94). Increasing evidence, now including successful male partner treatment strategies (95), suggests that BV itself may be sexually transmitted. Molecular concordance in bacterial occupancy between heterosexual partners further supports this conclusion (17, 96, 97). Multiple research studies in specific high-risk populations have also linked the psychological effects of chronic stress to patterns of high-risk sexual behaviors (45, 94), which are in turn linked to both BV and STI and often have strong links to other social determinants of health.

### Vaginal douching.

Associations between douching and BV are prominent in the literature (98). Douching has also been noted as a risk factor for subsequent development of PID and STIs (98, 99). A study of 72 individuals suggested that those with BV who douche were more likely to have a proinflammatory immune signature than those with BV who did not douche. Likewise, BV-associated douching was associated with higher cytokine levels compared with those without BV regardless of douching behavior (100). Together, these studies present circumstantial evidence that douching should be discouraged. In a small crossover study, douching cessation alone was *not* associated with significant changes in the representation of *Lactobacillus* or other common vaginal taxonomic groups (101). Larger and more comprehensive studies are needed to fully support recommendations, for example, from the American College of Obstetrics and Gynecology and Centers for Disease Control (102–104), that douching is unnecessary and may lead to poor health outcomes. In a large study of pregnant women, self-identified Black women had similar microbiomes whether they engaged in vaginal douching or not. However, douching White women tended to have microbiotas that looked more similar to Black women than nondouching White women, with higher numbers of *L*. *iners* and lower numbers of *L*. *crispatus*. Striking data show that White women who douched also had different socioeconomic indicators than nondouching White women, including significantly lower educational attainment, lower socioeconomic status, higher BMI, and more adverse life events (105). These observations support the interpretation that social factors are important drivers in BV irrespective of “race” and that douching may be a response to more severe BV symptoms. This does not exclude the possibility that douching also has independent effects on the vaginal microbiome.

## Microbial factors

Next, we discuss the roles played by BV bacteria and their coconspirators in directly causing infections or promoting them by engaging in disruptive behaviors that predispose the host to infection, including the provocation and manipulation of host responses (Figure 3).

### L. crispatus and L. iners.

Individuals with abundant *L*. *crispatus* (CST-I) have the lowest risk of vaginal infections with viruses, bacteria, and protozoan parasites (106, 107) compared with individuals with other state types, including CST-III (*L*. *iners* dominant). Although there may be confounders to consider, several lines of evidence suggest *L*. *crispatus* can have beneficial effects and that *L*. *iners* could be harmful or less protective than *L*. *crispatus* (107, 108). First, recent clinical trials that administered *L*. *crispatus* vaginally after antibiotic treatment for BV or recurrent UTI showed improvement in the recurrence rates of these conditions (109, 110). As well, some in vitro studies showed *L*. *iners* cultures made less lactic acid compared with *L*. *crispatus* cultures, potentially creating a less microbicidal milieu. *L*. *iners* encodes a cholesterol-dependent pore-forming toxin (inerolysin) capable of disrupting vaginal epithelial cells (111, 112). Finally, longitudinal studies investigating microbiota dynamics found that *L*. *iners*–dominant communities more frequently displayed shifts to *Lactobacillus*-depleted microbial communities. Additional studies are needed to clarify whether and in what specific contexts *L*. *iners* may have pathogenic effects.

### Gardnerella may encourage virulence of other pathogens.

*Gardnerella* is found in the healthy vaginal microbiome. It is also one of the most abundant bacterial species in BV and continues to be at the center of debates about causal relationships. Since human studies most often report associations, they cannot be used to prove direct causation. However, evidence from mouse models indicates that the presence of *Gardnerella* in the vagina can enhance infection by other pathogens. For example, in nonpregnant mice, coinoculation with *Gardnerella* resulted in higher levels of ascending uterine infection by *Prevotella bivia* (*P*. *bivia*) (113). In a timed-pregnancy mouse model, coinoculation with *Gardnerella* enhanced not only vaginal colonization, but also ascending uteroplacental infection by the neonatal sepsis pathogen group B *Streptococcus* (114). Whether these phenotypes were due to a direct effect of *Gardnerella* on the bacterial pathogens themselves, or indirect via the host response, remains to be determined.

*Gardnerella* also promotes the pathogenesis of UTI by uropathogenic *E*. *coli* (UPEC) (115, 116). *Gardnerella* is routinely found in urine collected from women, including samples collected by catheterization (117). In a mouse model, *Gardnerella* did not colonize the urinary tract. However, even short-lived transurethral exposures resulted in a transcriptional signature of inflammation and urothelial turnover (118, 119). *Gardnerella* exposure also resulted in evidence of kidney injury (higher serum creatinine) due to the proinflammatory effects of IL-1R signaling. In a separate model, *Gardnerella* was administered to mice after resolution of an experimental UPEC UTI. This secondary exposure to *Gardnerella* triggered the emergence of UPEC from quiescent intracellular reservoirs that had formed during the initial infection, resulting in recurrent UTI (116, 120), potentially due to disruption of the urothelial barrier by *Gardnerella*-induced exfoliation. In the UPEC recurrent UTI model, *Gardnerella* exposure was also associated with a greater likelihood of severe kidney abscess and splenomegaly with evidence of transient bacteremia (120).

### Growth synergy.

Several studies suggest synergistic growth or metabolism may contribute to BV and drive associations with other infections. For example, *Gardnerella vaginalis* (*G*. *vaginalis*) enhances *A*. *vaginae* viability in vitro (121). As well, *Trichomonas vaginalis* (*T*. *vaginalis*) inhibited the growth of *Lactobacillus*, but not BV, bacteria in an in vitro vaginal ectocervical infection model (122). In addition, *P*. *bivia* and *G*. *vaginalis* exhibited growth synergy via production and utilization of ammonia, respectively (123). *Peptostreptococcus anaerobius* growth was aided by amino acids produced by *P*. *bivia* (124, 125). Another form of metabolic interaction is the cross-species feeding of sugars scavenged from the host. Glycan chains with terminal sialic acids are abundant on cell surface and secreted glycoproteins at mucosal surfaces, including the vagina. A hallmark of BV is sialidase activity in cervicovaginal fluid, which is produced by BV bacteria including *Gardnerella* (126). Cooperation exists between sialidase-producing bacteria that liberate sialic acids, with pathogens like *F*. *nucleatum* that lack a sialidase but can transport and metabolize the monosaccharide (76). Mutant *F*. *nucleatum* incapable of sialic acid catabolism were less capable of colonizing the sialidase-positive mouse vagina.

*Chlamydia* is a tryptophan auxotroph. IFN-γ mediates protective immunity to *Chlamydia* infection by upregulating host indoleamine 2,3-dioxygenase (IDO1) (127), which catabolizes tryptophan, reducing its availability. *C*. *trachomatis* circumvents this by converting exogenous indole into tryptophan. Indole is present in vaginal fluid from women with BV (128), with CST-IV microbiota being associated with low levels of vaginal tryptophan and higher abundance of indole-producing bacteria (129). Furthermore, supernatants from indole-producing *Prevotella* strains, but not from indole-negative strains, promoted growth of tryptophan-starved *C*. *trachomatis* and rescued them from IFN-γ-mediated inhibition (130).

These data illustrate how dynamic commensal and synergistic relationships between bacteria may be driving the polymicrobial consortia we recognize as BV and creating niches for opportunistic pathogens to take root.

### Bacterial proximity and damage to the vaginal epithelium.

The epithelium is a physical barrier contributing to innate defense against invading pathogens. One characteristic feature of BV is the presence of an adherent, *Gardnerella* biofilm on the surface of vaginal epithelial cells. The biofilm is believed to contribute to antimicrobial tolerance and BV recurrence (131, 132). Recent studies indicate that the proximity of BV bacteria to the epithelium and their ability to attack the surface glycans with secreted glycosidases leads to a depletion of the protective glycocalyx (133) and may leave the epithelium more prone to colonization and attack. Additional species of vaginal bacteria, protozoan pathogens, and viral pathogens can become embedded into the biofilm. Examples include other BV bacteria and other genital pathogens known to co-occur with BV, such as *Trichomonas vaginalis*, which incited more damage to the host epithelium together with *Gardnerella* (134, 135) and UTI pathogens like *E*. *coli* and *Enterococcus* *faecalis* (136). These interactions can have functional consequences. Physical interactions between urinary pathogens *E*. *faecalis* and *Actinomyces neuii* increased expression of *Gardnerella* virulence factors cytolysin (vaginolysin) and sialidase (137). *Gardnerella* biofilms were also shown to be capable of retaining and then later releasing infectious *Chlamydia trachomatis* elementary bodies in vitro (138). Conditioned media from *A*. *vaginae–*infected endothelial cells enhanced HIV transepithelial migration, and this effect was significantly associated with the level of MMP activity (139). Treatment with MMP10 was sufficient to disrupt the endocervical barrier in vitro.

In another recent example, *N*. *gonorrhoeae* evaded immune detection by relying on damage caused to epithelial cells by *Gardnerella*. The gonococcus is known to scavenge activated sugars from the host and attach them to its own cell surface, preventing the host complement system from attacking (reviewed in ref. 140). In vitro models showed that *N*. *gonorrhoeae* incorporates more sialic acid into its protective coating when purified recombinant *Gardnerella* vaginolysin is present to liberate intracellular sources of the activated sialic acid intermediate, CMP-N-acetylneuraminic acid (141). Therefore, *Gardnerella*-aided glycan mimicry could be contributing to the association between BV and *N*. *gonorrhoeae* infection. Together, these studies point to the importance of colonization and damage to epithelial cells as targets for new treatment modalities to fight BV and its comorbid infections.

### BV-associated immune signatures impact viral infection.

BV has been associated with higher risks of HIV acquisition and HPV progression. Some studies have reported higher levels of proinflammatory cytokines, including IL-1β, IL-6, and TNF-α, in BV. These cytokines can stimulate HIV-1 infection by activating the long-terminal repeat promoter region of the HIV-1 NF-κB pathway, suggesting that they could play a role in the link between BV and HIV. In a study of 62 women at high risk for HIV and 33 low-risk individuals acting as controls (142) secretory leukocyte protease inhibitor (SLPI) levels were lower in women with BV and levels of the chemokine RANTES (CCL5) were higher in the high-risk group. There was a decrease in RANTES levels after BV treatment compared with paired pretreatment samples (142), suggesting that the microbiome influences RANTES production.

Compared with women with *Lactobacillus*-dominant vaginal microbiomes, women with BV or CST-IV were shown to harbor higher levels of mucosal activated CD4^+^ T cells targeted by HIV (143). In contrast, the levels of protective γδ 1 (GD1) T cells were lower, and HIV target GD2 T cells were higher in women with abnormal vaginal flora (Nugent score >3) (144). However, another study found no significant associations between microbial community structure and cervical T cell counts or markers of adaptive immune activation (145, 146). Another recent study investigated Langerhans cells (LCs) in the vagina, which are known to capture and degrade HIV-1 and serve as antigen-presenting cells (147, 148). These functions were inhibited in the context of inflammation, where LCs instead transmitted HIV-1 to CD4^+^ T cells (148). When LCs were exposed to 11 species of UV-inactivated vaginal bacteria in vitro, only *P*. *timonesis* enhanced LCs uptake of HIV-1, but the virus bypassed autophagic clearance and LCs remained infectious for several days, with enhanced transmission to U87-CD4-CCR5 cells (149).

Associations between vaginal inflammatory mediators are also believed to mediate interactions between BV and HPV. The invasive cervical carcinoma outcomes in patients who were positive for HPV were different between women with *Lactobacillus-*dominant microbiota (positive association with MIP-1β and IL-2) or non-*Lactobacillus-*dominant microbiota (positive association with IP-10, RANTES, Flt-3L, IL-4, and sCD40L) (150). Only IL-36γ was positively associated with invasive cervical carcinoma in both vaginal microbiome groups.

### Immunomodulatory glycan-binding proteins in BV-associated trichomoniasis.

There is also evidence that changes to immune signatures in BV can impact parasitic infections. There is a significant association between BV and trichomoniasis. *T*. *vaginalis* cases with BV or intermediate Nugent scores expressed higher levels of galectin-9 and IL-1β and lower levels of galectin-3 compared with trichomoniasis in the absence of BV (151). An in vitro infection model implicated *P*. *bivia* as a causal factor in this relationship. In this model of in vitro vaginal epithelial infection, *T*. *vaginalis* synergized with its endosymbiont Trichomonasvirus (TVV) and BV bacteria to upregulate galectin-1, galectin-9, and inflammatory cytokines. *P*. *bivia* downregulated *T*. *vaginalis*+TVV–induced antiviral response (IP-10 and RANTES). *P*. *bivia* downregulated galectin-3 but led to higher expression of galectin-1, galectin-9, and IL-1β. These results were consistent with the clinical findings of mixed *T*. *vaginalis*–BV infections in the same study (151). Additionally, *A*. *vaginae* and *G*. *vaginalis* amplified epithelial cell proinflammatory responses to the *Trichomonas* virulence factors LPG/CPI-GC and TVV, whereas *P*. *bivia* suppressed TV/TVV-induced chemokines (122).

## Host genetics and other intrinsic factors

Finally, we discuss the role of factors that are intrinsic to the host, for example, those based on host age, genetics, or immunity, in the associations between BV and infection.

### The vaginal microbiota over the lifespan.

The vaginal microbiome shifts throughout the lifespan in response to intrinsic changes in hormonal composition, for example, at menarche; during menstrual cycles (16), pregnancy, and postpartum (152, 153); and in menopause (154). The physiology of the vaginal epithelium is highly responsive to estrogen and often does not support *Lactobacillus* species in menopause. However, vaginal estrogen replacement is highly effective at restoring the epithelium to a state that can support *lactobacilli* (155). Early studies described reproductive-age (premenopausal) women as being most likely to carry *L*. *crispatus–*dominant (CST-I), *L*. *iners–*dominant (CST-III), or the “diverse”, non-*Lactobacillus*-dominant (CST-IV) microbiotas. The diverse CST-IV microbiota has begun to be characterized with more intention. The CST-IV-B pattern is similar to BV, with high levels of *Gardnerella* and *Fannyhessea*. In postmenopausal women, another pattern reveals a substantial abundance of *Streptococcus* and *Prevotella* (CST-IV-A) (154). Other studies confirm that *Lactobacillus*-dominant microbiomes are less often harbored by postmenopausal women (reviewed in ref. 156). A systematic review of 13 studies reported postmenopausal women with an overall BV (by Nugent or Amsel criteria) prevalence of 16.9%, ranging between 2% and 57% (157). However, there is debate about what should be called “BV” in postmenopausal women, since the community of microbes is different than that seen in premenopausal women and is not always associated with symptoms (18, 156). Recently, the pregnancy-to-postpartum transition vaginal microbiome has also been characterized as having higher abundance of the genera *Streptococcus* and *Prevotella* (152), raising the possibility that understanding this pattern may yield benefits for women across the hormonal stages of life.

### Genomics and genetics.

Several SNPs have been linked with BV in GWAS (Supplemental Table 1; supplemental material available online with this article; https://doi.org/10.1172/JCI184322DS1). Given the known roles of the identified proteins, receptors, and proteoglycans in infection (see below), it is plausible that these polymorphisms impact the risk of BV and other infections. Although little follow-up has been done so far, the results of these studies suggest several encouraging theoretical mechanisms linking BV to risks of infection.

### Syndecans.

Syndecans are a class of glycosylated proteins that are heavily modified with heparan sulfate (HS) found on the vaginal and uterine epithelium (158). One GWAS of 686 women with or at risk of HIV identified several SNPs that were associated with BV status in all 4 human syndecan genes (*SDC1-4*) (159). Self-reported race and ethnicity, as well as geographical information and “ancestry-informed” molecular markers, were used to “estimate” ancestry and to identify 10 SNPs in *SDC1*, -*2*, and -*4* tied to a higher risk of BV within each group of individuals studied. Different numbers of individuals studied could have different statistical power to detect differences in SNP frequency between those with and without BV. Nevertheless, the study identified BV-associated syndecan SNPs in all ancestral groups studied. SNPs in syndecans have also been found to be associated with STIs. Many STI pathogens adhere to HS proteoglycans as a strategy for infection (160–163). It is hypothesized that these SNPs could affect HS-binding by BV-associated microbes and pathogens of other STIs like chlamydia, HIV, and HPV.

### Mannose-binding lectins.

Another group of implicated proteins are mannose-binding lectins (MBLs), pattern recognition receptors important in the complement pathway that recognize and flag pathogens for downstream immune processing (164). The underlying mechanisms are not yet understood, but it is thought that disruption of MBL-pathogen binding could play a role in infection progression or recurrence. In a study of 171 Kenyan women, SNPs in the MBL (*MBL2*) region were significantly associated with decreased abundance of *L*. *iners*, increased abundance of *Gardnerella*, and greater α-diversity (CST-IV) (164). Studies in cohorts of Indian and Brazilian women also reported associations between *MBL2* polymorphisms and increased risk of BV, but other studies in Italian women failed to find associations between MBL SNPs and BV (165–167).

### IL-1 signaling.

SNPs in IL-1α, IL-1β, and IL-1ra have also been associated with disease. IL-1α and IL-1β are important cytokines that mediate proinflammatory responses via the ubiquitously expressed IL-1 receptor. In one study of US women, carriage of IL-1 receptor antagonist allele 2 (IL1RN*2) was significantly associated with a dampened IL-1β response in the presence of *G*. *vaginalis* and Gram-negative rods, along with a reduced rate of spontaneous preterm birth (168). The hypothesis that imbalances in either direction of the inflammatory response could influence risks for preterm birth is also supported by studies linking preterm birth with higher levels of maternal proinflammatory IL-1β, IL-6, TNF-α, and with SNPs in IL-1β (169–171). During infection, IL-1β and IL1-ra are often coexpressed and mediate an adequate immune response by competing for binding to IL-1R. SNPs in IL1RN*2 could be promoting higher expression of IL1-ra, reducing the IL-1β–mediated inflammation and lowering the chance of preterm birth, as seen in ref. 168. In a small cohort of pregnant women, those with BV had higher concentrations of IL-1α relative to healthy individuals acting as controls, and IL-1α correlated with levels of endotoxin in cervical mucus and vaginal fluid (172).

### TLRs.

SNPs in TLR4 and TLR2 have also been hypothesized to be creating imbalanced immune responses in the context of infection and disease. In one study of pregnant women, the TLR4 896G polymorphism was linked with a BV-like microbiome and, surprisingly, 896G carriers did not exhibit the same elevated IL-1 or IL-1ra levels seen in 896A homozygotes despite both comparison groups having BV-associated bacteria (168). However, only approximately 10% of individuals in the study carried the 896G allele. While several studies have failed to find TLR4 in the vaginal epithelium, its expression has been reported in the endocervix, endometrium, and fallopian tubes (122, 171, 173). In women with clinical PID, SNPs in *TLR2* were modestly associated with the presence of cervical *A*. *vaginae* (174), further supporting the hypothesis that polymorphisms in TLRs may promote differential sensitivities or responses to microbial products and help to explain differential health outcomes experienced by patients.

## Summary and outlook

In closing, reported links between BV and other infections are many. Ongoing research continues to map the landscape and topography of mechanisms that underlie these connections. Clear parallels between the risk factor profiles of BV and infections, including socioeconomic indicators and associated psychosocial stress, sexual behavior, nutrition, smoking, douching, and contraceptive choices may explain some instances of BV and infection co-occurrence. Progress has also been made in identifying single nucleotide genetic variants mostly associated with host immunity that are linked with BV. How the identified SNPs function in experimental disease models remains to be determined. We emphasize that BV is not “just” a lack of protective bacteria predisposing a person to infection. As discussed above, mounting evidence indicates that BV bacteria can actively inflict harm on the vaginal mucosa and can trigger host and microbial responses linked with susceptibility to other infections. A network of synergistic, symbiotic, and opportunistic interactions is beginning to emerge between the many microbes of the female urogenital system and the host environment. This interconnected biome consists of individual members in functional relationships studied mostly in a pairwise fashion. Additional epidemiological, clinical, and molecular research is needed to support or refute further pairwise and higher-order interaction studies to identify the links supporting pathogen outgrowth and virulence. New bacterial genetic systems will be a crucial step forward to understand the mechanisms. Experimental models are needed that can also integrate the impacts of severe psychosocial stressors, relevant contexts of host immunity, and specific combinations of microbes to better define how these complex factors influence the development of infections in the setting of BV.

## Supplementary Material

Supplemental data

## Figures and Tables

**Figure 4:**
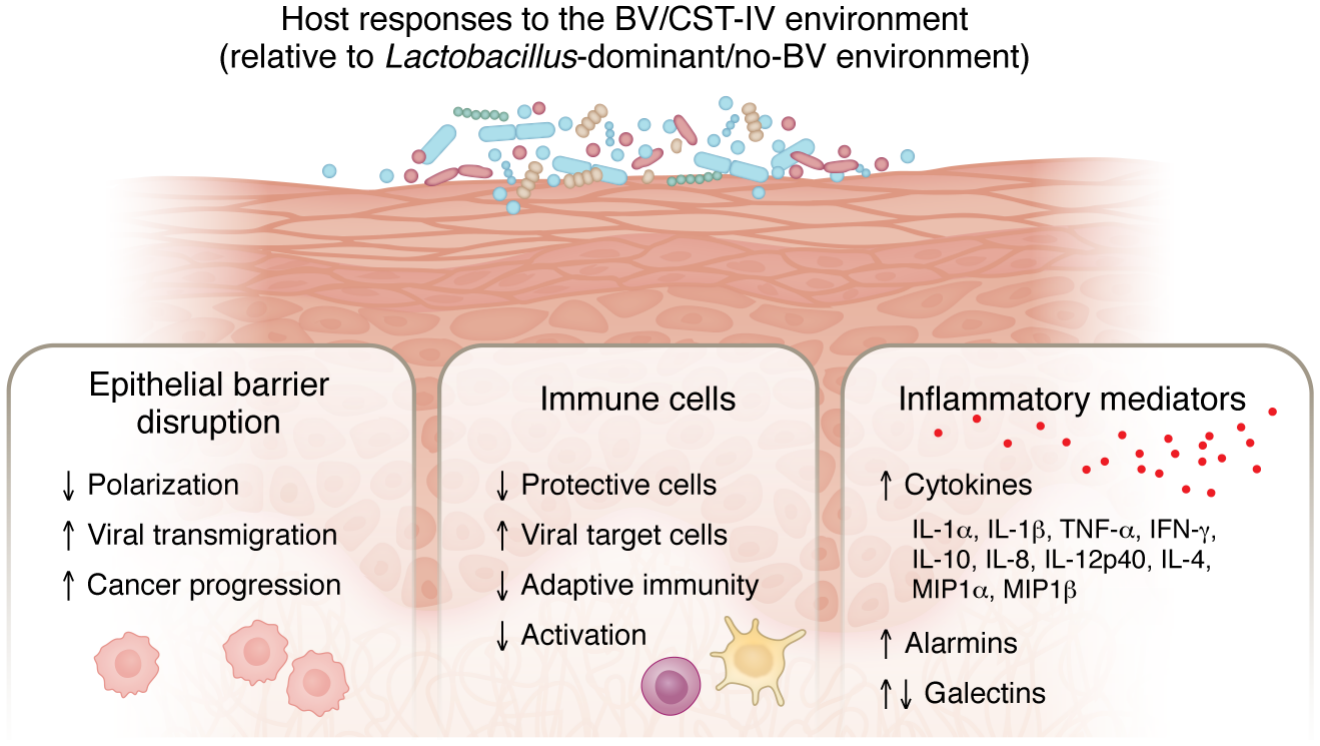
Host responses in the setting of BV versus Lactobacillus-dominant microbiota are distinct.

**Table 1:**
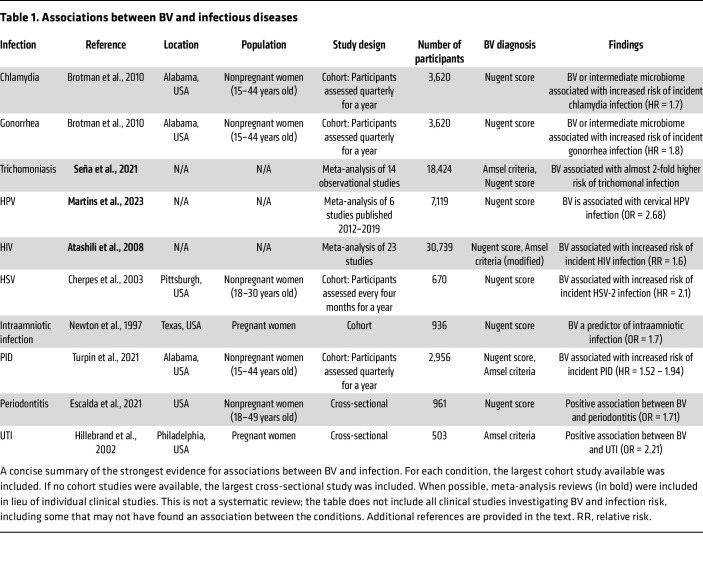
Associations between BV and infectious diseases

## Version 1 06/02/2025

Electronic publication
