# Supplementary material for: Social, microbial, and immune factors linking bacterial vaginosis and infectious diseases
Source: J Clin Invest. 2025 Jun 2;135(11):e184322. doi: 10.1172/JCI184322 (PMC12126241; doi:10.1172/JCI184322)
Supplement: Supplemental data [file jci-135-184322-s135.pdf]

Supplemental Table 1: Single Nucleotide Polymorphisms Associated with BV and Inflammatory Phenotypes

Non-comprehensive list of studies that found associations or lack thereof between particular SNPs in Syndecans, Interleukins, Mannose Binding Lectins, or Toll-like Receptors and various types of observations such as increased or decreased risk of disease, presence of specific vaginal microbes, or other outcomes. For context, we note that most “Hispanic” and “Black” individuals share substantial European ancestry. Recent African ancestry contribution in self-identified African Americans ranges widely. (173,174) Considering the extremely rich genetic diversity in Africa, most genomic variants outside Africa represent but a fraction of those existing in Africa. Thus, people with more recent, even partial African ancestry, are found to carry SNP variants not found in people outside Africa.

Gene	SNP	Association	Comparison Groups	Source	PMID
SDC1	rs4666298	Increased BV risk	SNP carriers vs non-carriers of African Ancestry	Murphy et al.	38720636
	rs74346376	Increased BV risk	SNP carriers vs non-carriers of African-Ancestry		
SDC2	rs2439525	Decreased BV risk	SNP carriers vs non-carriers of Hispanic-Ancestry (west & east coast participants)		
	rs2582839	Decreased BV risk	SNP carriers vs non-carriers of African & Hispanic-Ancestry (west coast participants)		
	rs724235	Decreased BV risk	SNP carriers vs non-carriers of African-Ancestry		
	rs895034	Decreased BV risk	SNP carriers vs non-carriers of African-Ancestry		
SDC3	rs12085929	Increased BV risk	SNP carriers vs non-carriers of European-Ancestry		
	rs4949320	Increased BV risk	SNP carriers vs non-carriers of European & Hispanic-Ancestry (west coast participants)		
	rs56090580	Decreased BV risk	SNP carriers vs non-carriers of Hispanic-Ancestry (east coast participants)		
SDC4	rs1008953	Decreased BV risk	SNP carriers vs non-carriers of African-Ancestry		
	rs2228384	Increased BV risk	SNP carriers vs non-carriers of Hispanic-Ancestry (west coast participants)		
	rs2267868	Increased BV risk	SNP carriers vs non-carriers of African-Ancestry		
	rs2284278	Increased BV risk	SNP carriers vs non-carriers of African-Ancestry		
TLR1	rs200232721	Decreased BV risk	SNP carriers vs non-carriers of African-Ancestry		
	rs200232721	Increased BV risk	SNP carriers vs non-carriers of European-Ancestry		
TRL2	rs1816702	Increased BV risk	SNP carriers vs non-carriers of African-Ancestry		
TLR4	rs10759930	Increased BV risk	SNP carriers vs non-carriers of European-Ancestry		
TLR9	rs187084	Increased BV risk	SNP carriers vs non-carriers of Hispanic-Ancestry (west coast participants)		

IL1A	rs2071376	Increased BV risk	SNP carriers vs non-carriers of European & Hispanic-Ancestry (east coast participants)		
IL6	rs1880243	Increased BV risk	SNP carriers vs non-carriers of African-Ancestry		
	rs2069840	Increased BV risk	SNP carriers vs non-carriers of European-Ancestry		
IL1B	rs3136558	Decreased BV risk	SNP carriers vs non-carriers of African-Ancestry		
	rs10169916	Decreased BV risk	SNP carriers vs non-carriers of African-Ancestry		
	rs12621220	Decreased BV risk	SNP carriers vs non-carriers of African-Ancestry		
IL2	rs2069771	Decreased BV risk	SNP carriers vs non-carriers of African-Ancestry		
IL1RN	rs62158853	Decreased BV risk	SNP carriers vs non-carriers of European-Ancestry		
MBL2	rs7084554	Carriage of C allele	Patients with recurrent vulvovaginal infection vs healthy control	Kenia et al.	31080578
	rs36014597	Carriage of G allele	Patients with recurrent vulvovaginal infection vs healthy control		
	rs7084554	TC genotype	Patients with vulvovaginal candidiasis vs healthy control		
			Patients with mixed infections vs healthy control		
		Carriage of C allele	Patients with BV vs healthy controls		
			Patients with mixed infections vs healthy control		
	rs36014597	AG genotype	Patients with BV vs healthy controls		
			Patients with vulvovaginal candidiasis vs healthy control		
			Patients with mixed infections vs healthy control		
		Carriage of G allele	Patients with BV vs healthy controls		
		Carriage of G allele	Patients with vulvovaginal candidiasis vs healthy control		
		Carriage of G allele	Patients with mixed infections vs healthy control		
IL-1ra	IL1RN*2 (IL-1 receptor antagonist allele 2)	Lower prevalence of IL1RN*2 carriage in black women	All IL1RN*2 carriers vs All IL1RN*2 non-carriers	Genc et al.	15507961
		Lower rate of spontaneous pre-term birth			
		Increased vaginal pH in IL1RN*2 carriers			
		Lower levels of Lactobacilli colonization	Black women who are IL1RN*2 carriers vs non-carriers		

		Increased levels of gram negative rods			
		Increased rate of BV (Nugent score >7)			
		Reduced IL-1B response	All IL1RN*2 carriers with abnormal microflora vs all IL1RN*2 non-carriers with abnormal microflora		
TLR4	rs4986790	Increased G. vaginalis abundance	TLR4 896G carriers with BV vs TLR4 896A homozygotes with BV	Genc et al.	15358455
		Increased vaginal IL-1B			
		Increased vaginal IL-1ra			
MBL2	Codon 54 allele B	Higher rate of allele B carriage	Recurrent BV vs Acute BV	Giraldo et al.	17470593
		Higher rate of allele B carriage	Individuals with recurrent Candidiasis vs healthy controls		
MBL2	rs11003099	Decreased *L. iners* abundance	SNP carriers vs non-carriers	Mehta et al.	32723796
	rs114589489	Increased G. vaginalis abundance	SNP carriers vs non-carriers		
	rs7077650	Increased odds of CST-IV	SNP carriers vs non-carriers		
MBL2	Codon 52	No Association	Recurrent BV vs Healthy Controls	Milanese et al.	18211540
			Recurrent vulvovaginal candidiasis vs Healthy Controls		
	Codon 54	No Association	Recurrent BV vs Healthy Controls		
			Recurrent vulvovaginal candidiasis vs Healthy Controls		
	Codon 55	No Association	Recurrent BV vs Healthy Controls		
			Recurrent vulvovaginal candidiasis vs Healthy Controls		
TLR2	rs11938228	Increased likelihood of having cervical *Atopobium vaginae*	CC genotype vs AC genotype for TLR2 -1733 C>A	Taylor et al.	29286178
	rs1898830		AA genotype vs AG genotype for TLR2 -616A>G		
IL-1B	rs1143634	Lower risk of Preterm Birth	IL-1B +3953C>T carriers vs IL-1B +3953C>T non-carriers	Schmid et. al	22884587
